# Revisiting California’s Supervising Physician-to-Physician Assistant Ratio Requirement: An Urgent Call to Action

**DOI:** 10.7759/cureus.60926

**Published:** 2024-05-23

**Authors:** Vasco D Kidd

**Affiliations:** 1 Orthopedic Surgery, University of California Irvine School of Medicine, Irvine, USA

**Keywords:** physician collaboration or supervision, hospital administration, workforce planning, ratio requirements, primary care, public health, healthcare reform, patient access, california, physician assistant

## Abstract

California like many other states is facing a severe shortage of primary care physicians and access to primary care is uneven across the state. It is well documented that California has the highest number of designated primary care health professional shortage areas in the country. Although physician assistants (PAs) and nurse practitioners (NPs) are estimated to make up a large portion of California’s primary care workforce by 2030, outdated and unnecessary statutory requirements such as the physician-to-PA supervision ratio requirement represent a practice barrier in expanding access to care. Other states have either eliminated or revised their physician-to-PA supervision ratios in favor of expanding access to health care services. Therefore, this editorial represents a call for coordinated actions from local and state entities to address California's outdated physician-to-PA supervision ratio requirement. NPs are mentioned briefly as they have achieved a pathway to full practice authority in California.

## Editorial

According to the California Health Care Foundation (CHCF), “California is not producing enough healthcare workers to meet the needs of a growing, aging, and rapidly diversifying population”. Unfortunately, only half of the state’s primary care needs were met in 2023 [1]. Factors exacerbating California’s existing workforce shortage may be related to geographic scarcity or maldistribution of health professionals [2]. In the next few years, physician assistants (PAs) and nurse practitioners (NPs) will comprise nearly half of the state's full-time equivalent primary care clinicians by some estimates; however, many Californians will continue to have insufficient access to a primary care provider [3]. 

One of the ways to help improve access to primary care and other specialties is to repeal or revise the numerical limit on the number of PAs a physician may supervise in California. According to Business and Professions Code (BPC) 3516 (b), a physician and surgeon shall not supervise more than four PAs at a time. The 1:4 supervision ratio applies to PAs who provide direct patient care and prescribe medication. This law has been in effect since 2008. However, during the coronavirus disease 2019 (COVID-19) pandemic, the Department of Consumer Affairs (DCA) issued a temporary state-level waiver on 3/30/2020, suspending the ratio requirement of four PAs for any supervising physician. The waiver was widely celebrated by many organizations including the California Medical Association (CMA) which posted an article on their website that reads in part: “Physicians can now supervise the number of NPs and PAs they can competently and confidently supervise without a statutory ratio in place” [4]. ​​Although the waivers were rescinded following termination of the State of Emergency on 2/28/2023, the California Physician Assistant Board (PAB) did not receive any complaints and/or concerns associated with a physician supervising more than four PAs simultaneously during the nearly three-year temporary suspension of the ratio requirement. This is not surprising, as there is no publicly available data or evidence underpinning the need for maintaining the current physician-to-PA supervision ratio in California. In fact, 20 other states have eliminated strict physician-to-PA supervision ratio requirements (Table 1) [5]. To my knowledge, there have been no peer-reviewed publications that have examined whether removing or increasing legislated PA supervision ratios has led to improved access to healthcare services. 

**Table 1:** No maximum supervision PA ratios PA: Physician assistant

Number	States with no supervision PA ratio requirement
1	Alaska
2	Connecticut
3	Idaho
4	Maine
5	Massachusetts
6	Michigan
7	Mississippi
8	Montana
9	New Hampshire
10	New Mexico
11	North Carolina
12	North Dakota
13	Oregon
14	Rhode Island
15	Tennessee
16	Utah
17	Vermont
18	West Virginia
19	Wisconsin
20	Wyoming

Although 29 states maintain physician-to-PA supervision ratios, 45% (13/29) of those states within the last seven years have increased the number of PAs a single physician may supervise or collaborate with at any one time (Table 2) [5-6]. The number ranges from 4 to 10 PAs. It is equally important to note that some states with physician-to-PA supervision ratios have waived these requirements for practices serving medically underserved areas or those providing care in a hospital setting. Alabama was not listed in Table 2 since PA supervision cannot exceed the approved cumulative work time per week. Alabama has enhanced a physician's ability to supervise from 160 hours (four full-time equivalent PAs per week) to now 360 hours per week (nine full-time equivalent PAs). 

**Table 2:** Side-by-side comparisons of states with maximum PA supervision ratio requirements (2017-2024) PA: Physician assistant *A physician may petition for a waiver to exceed the maximum ratio limit. 2024 Physician/PA ratio data for each state from the National Conference of State Legislatures (NCSL)

States	Maximum PA supervision ratio requirements (seven-year trend)
Arizona	Increased the physician/PA ratio from 1:4 to 1:6
Colorado	Increased the physician/PA ratio from 1:4 to 1:8
Florida	Increased the physician/PA ratio from 1:4 to 1:10
Hawaii	Increased the physician/PA ratio from 1:2 to 1:4
Illinois	Increased the physician/PA ratio from 1:5 to 1:7
Kansas	Increased the physician/PA ratio from 1:3 to 1:5
Louisiana	Increased the physician/PA ratio from 1:4 to 1:8
Missouri	Increased the physician/PA ratio from 1:3 to 1:6
Ohio	Increased the physician/PA ratio from 1:3 to 1:5
Oklahoma	Increased the physician/PA ratio from 1:4 to 1:6
Pennsylvania	Increased the physician/PA ratio from 1:4 to 1:6
South Carolina*	Increased the physician/PA ratio from 1:3 to 1:6
Washington*	Increased the physician/PA ratio from 1:5 to 1:10

I would be remiss if I did not mention that decisions related to the number of PAs to supervise or collaborate with is based on a number of factors including but not limited to practice size, expansion of services, patient wait times, clinic space, the PA's education, training, and experience. That said, many states have repealed or made significant revisions to their physician-to-PA supervision ratios over the last several years which is emblematic of a larger trend to improve access to care benefiting providers and patients alike. 

A call to action

California should consider either removing or increasing its outdated legislated physician-to-PA supervision ratio as other states have done to improve access to healthcare services especially in primary care where the state faces a projected shortfall of nearly 9,000 primary care clinicians between 2025 and 2030 [3]. The statewide primary healthcare shortage will likely exacerbate healthcare disparities especially among black and brown communities which are vulnerable to poor health outcomes. It is well known that unhindered access to timely primary care services is necessary to improve population health, reduce health disparities, and advance health equity. Prior research has demonstrated that PAs are more likely to practice in areas where there are health professional shortage areas (HPSAs) especially in California [7]. Around 33% of Californian's population is located in a primary care area (HPSA) [8]. Therefore, addressing the state’s outdated physician-to-PA ratio is a positive step forward for improving patient care access. 

There are two options to repeal or revise California's physician-to-PA ratio. The first option is to propose a change to the physician-to-PA supervision ratio during the Physician Assistant Board's (PAB) upcoming sunset review and to raise it as a new issue for consideration by the Legislature. Any approved legislative changes to the physician-to-PA supervision ratio through the sunset review process would be reflected in the PAB’s sunset bill, which would take effect on January 1st, 2026. The other option is to defer to constituent organizations and/or other stakeholders to work with a senator or assembly member to help author a bill to change the physician-to-PA supervision ratio. However, this option seems unnecessary due to the cost, time, and resources required to pass a bill to address one code section. 

Although some may argue against altering the longstanding physician-to-PA supervision ratio, there is no clear empirical evidence that California’s legislated physician-to-PA supervision ratio reduces healthcare costs, improves patient safety, or supports better patient outcomes. Additionally, emerging research suggests that removing restrictive laws and regulations to PA practice does not increase overall risks to patients or lead to increased risk of medical malpractice [9]. Lastly, a recently published national straw poll found that 91% of United States adults say that PAs provide safe and effective care, and these consumers support updating PA laws to ensure healthcare systems fully utilize their healthcare workforce [10].

In closing, the statewide demand for primary care services combined with a shortage of primary care physicians should serve as a wake-up call that we cannot continue with a one-size-fits-all physician-to-PA supervision ratio. Expanding practice autonomy to allow California physicians to determine at the practice level the adequate number of PAs they can competently supervise may improve access to primary care services and other specialties. 
